# Diagnosis of Atopic Dermatitis: Mimics, Overlaps, and Complications

**DOI:** 10.3390/jcm4050884

**Published:** 2015-05-06

**Authors:** Elaine C. Siegfried, Adelaide A. Hebert

**Affiliations:** 1 Saint Louis University, Cardinal Glennon Children’s Hospital, 1465 South Grand Avenue, St. Louis, MO 63104, USA; 2 University of Texas-Houston Medical School, 6655 Travis, Suite 980, Houston, TX 77030, USA; E-Mail: Adelaide.A.Hebert@uth.tmc.edu

**Keywords:** atopic dermatitis, differential diagnosis, eczema, adult, adolescent, child, eczema herpeticum, eczema coxsackium, immunodeficiency, seborrheic dermatitis, psoriasis

## Abstract

Atopic dermatitis (AD) is one of the most common skin diseases affecting infants and children. A smaller subset of adults has persistent or new-onset AD. AD is characterized by pruritus, erythema, induration, and scale, but these features are also typical of several other conditions that can mimic, coexist with, or complicate AD. These include inflammatory skin conditions, infections, infestations, malignancies, genetic disorders, immunodeficiency disorders, nutritional disorders, graft-*versus*-host disease, and drug eruptions. Familiarity of the spectrum of these diseases and their distinguishing features is critical for correct and timely diagnosis and optimal treatment.

## 1. AD Diagnosis

Atopic dermatitis (AD) is the most common chronic skin disease in children, typically presenting in patients less than two years of age. Prevalence is highest among black children [1] with age-related gender variation. A slight predominance has been documented in male pre-school-aged children and in adult females [2].

**Figure 1:**
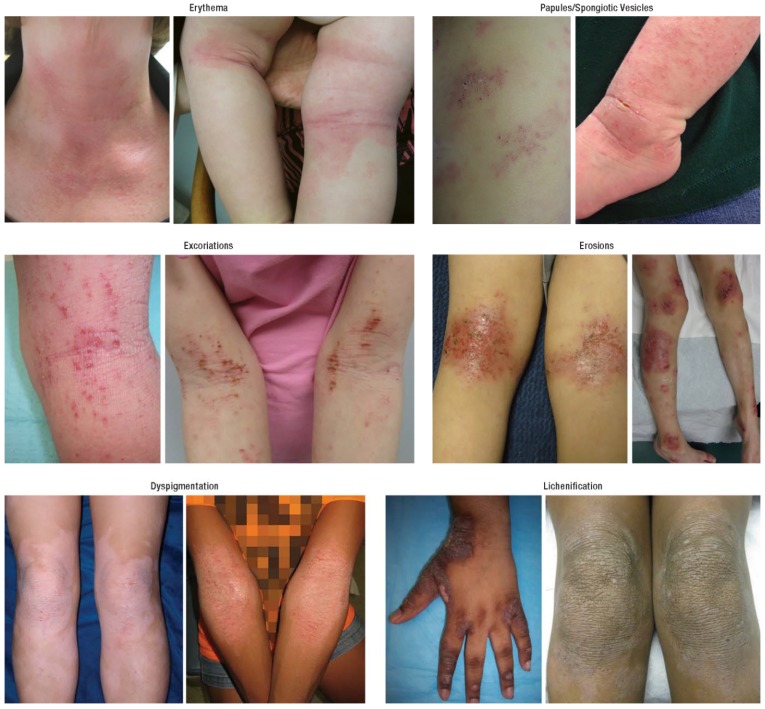
Atopic dermatitis: clinical features.

The diagnosis is based on age-specific clinical criteria that include pruritus and chronic or relapsing spongiotic dermatitis involving the face, trunk, and/or extensor extremities in infants, flexural surfaces like the wrists/ankles and antecubital/popliteal fossae in children, or the hands in adults (Table 1). The “diaper area”/groin and axillae are typically spared. Generalized xerosis is a ubiquitous feature, frequently with coarse ichthyosiform scale and palmoplantar hyperlinearity. Skin lesions are typically diffuse and very pruritic. AD is characterized by interval flares, often without obvious triggers. Acutely flaring AD features erythema, edema, and scale, often with multiple and widespread excoriations (Figure 1). Papular follicular changes are more prominent in darker skin types. In more severe cases, fine vesicles/papules are obvious, with serous drainage and crusting. Lichenification and dyspigmentation are chronic changes, and are more prominent in darker skin types. Frequent comorbidities include sleep impairment, psychiatric and mood disorders, asthma, allergic rhinitis, and allergic conjunctivitis. Eosinophilic gastroenteritis and celiac disease are also frequent comorbidities of AD; however, there is currently no evidence to support implementing food elimination diets (including gluten-free diets) in the absence of suggestive signs or symptoms.

**Table 1:** American Academy of Dermatology (AAD) Diagnostic Criteria for Atopic Dermatitis. AD indicates atopic dermatitis; IgE, immunoglobulin E.

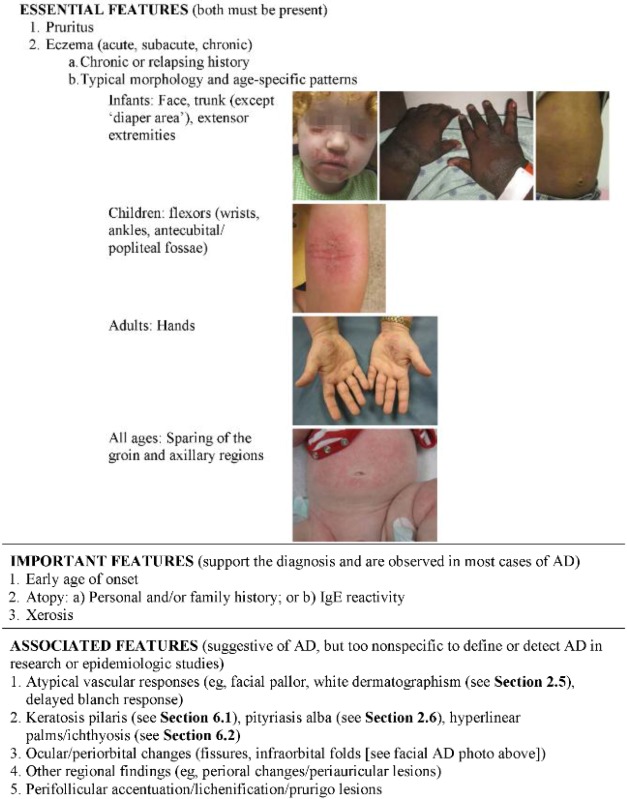

Adapted with permission of American Academy of Dermatology, Inc., from: Eichenfield, L.F.; Hanifin, J.M.; Luger, T.A.; Stevens, S.R.; Pride, H.B. Consensus conference on pediatric atopic dermatitis. *J. Am. Acad. Dermatol*. **2003**; *49* (6):1088–1095; permission conveyed through Copyright Clearance Center, Inc.

Associated laboratory abnormalities include high levels of immunoglobin E (IgE) and circulating eosinophils, but pathognomonic biomarkers have not yet been identified, so diagnosis is based on personal/family history of atopy and physical examination to exclude other conditions. A number of mimics, overlaps, and complications of AD exist and these differ by age group (Table 2 and Table 3). Section 2, Section 3, Section 4, Section 5, Section 6, Section 7, Section 8 and Section 9 discuss how these conditions may be confused with, coexist with, and/or complicate AD. Blood and skin testing for food and environmental allergic triggers should only be considered for patients with suggestive signs temporally related to exposure (*i.e.*, urticaria, gastrointestinal or respiratory symptoms, anaphylaxis), or those with AD that is unresponsive to optimized treatment.

**Table 2:** Diagnosis of atopic dermatitis: common mimics, overlaps, and complications and relative prevalence by age group.

	Diagnosis	Relative Prevalence		
		Infants	Children	Adolescents/Adults
**Inflammatory Skin Conditions** (See Section 2)	seborrheic dermatitis	common	uncommon	common
	psoriasis	less common	less common	common
	nummular dermatitis	less common	common	less common
	contact dermatitis ^a^	common	common	common
	dermatographism ^a^	less common	common	common
	pityriasis alba ^a^	common	common	uncommon
	overlap (see Section 2.7)	common	common	common
**Infections** (See Section 3)	impetigo ^a^	common	common	less common
	secondary syphilis	rare	rare	rare
	molluscum dermatitis ^a^	common	common	less common
	eczema herpeticum ^a^	uncommon	uncommon	rare
	eczema vaccinatum ^a^	rare	rare	rare
	eczema coxsackium ^a^	emerging	emerging	rare
	viral exanthem	common	common	less common
	tinea (as AD mimic)	uncommon	uncommon	uncommon
	candidiasis	common	less common	less common
**Infestations** (See Section 4)	scabies (prevalence varies by region)	may be common	may be common	may be common
**Genetic Disorders** (See Section 6)	keratosis pilaris	less common	common	not common
	ichthyosis vulgaris ^a^	common	common	common
**Immunodeficiency Disorders** (See Section 7)	HIV/AIDS-related skin changes (prevalence varies by region)	less common	less common	may be common
**Other** (See Section 9)	drug eruptions	less common	common	common

^a^ Frequent complication of AD.

Accurate diagnosis is critical to optimal patient care. Standard approach to treatment begins with education regarding the chronic relapsing nature of AD, which is be best controlled by bland skin care and adequate amounts of topical corticosteroids (TCS) for flares [4,5], In addition, topical calcineurin inhibitors (TCIs) or mild-to-moderate TCS can be used for proactive maintenance treatment in moderate-to-severe chronic disease.

**Table 3:** Differential diagnosis of atopic dermatitis: Rare disorders by age group. Ig indicates immunoglobulin.

	Infants	Children	Adolescents/Adults
**Malignancies** (See Section 5)	● Letterer-Siwe Disease	● Letterer-Siwe Disease	● Letterer-Siwe Disease
	● cutaneous T-cell lymphoma	● cutaneous T-cell lymphoma	● cutaneous T-cell lymphoma
**Genetic Disorders** (See Section 6)	● X-linked recessive ichthyosis (males only)	● X-linked recessive ichthyosis (males only)	● X-linked recessive ichthyosis (males only)
	● lamellar ichthyosis		● lamellar ichthyosis
	● nonbullous ichthyosiform erythroderma	● nonbullous ichthyosiform erythroderma	● nonbullous ichthyosiform erythroderma
**Immunodeficiency Disorders** (See Section 7)	● Netherton syndrome	● IgA deficiency ● IgM deficienc	● IgA deficiency
	● STAT3 deficiency		
	● DOCK8 deficiency		
	● Wiskott-Aldrich syndrome (males only)		
	● Leiner Phenotype		
	○ severe combined immunodeficiency		
	○ Omenn syndrome		● IgM deficiency
	● hypohidrotic ectodermal dysplasia/*NEMO*		
	● autoimmune polyendocrinopathy-candidiasis-ectodermal dystrophy (APECED)		
**Nutritional Disorders** (See Section 8)	● cystic fibrosis	●	● dermatitis herpetiformis
	● phenylketonuria		
	● zinc deficiency		
	● biotin deficiency		
**Other** (See Section 9)	● maternal-fetal graft-*versus*-host disease	● graft-*versus*-host disease	● graft-*versus*-host disease

## 2. Inflammatory Skin Conditions

### 2.1. Seborrheic Dermatitis

Seborrheic dermatitis (SD) is a common inflammatory skin condition most prominent on sebum-rich skin of the scalp and face. Onset is related to hormone-driven sebum production during early infancy, which wanes along with prevalence in childhood and increases again in adolescence and adulthood.

**Figure 2:**
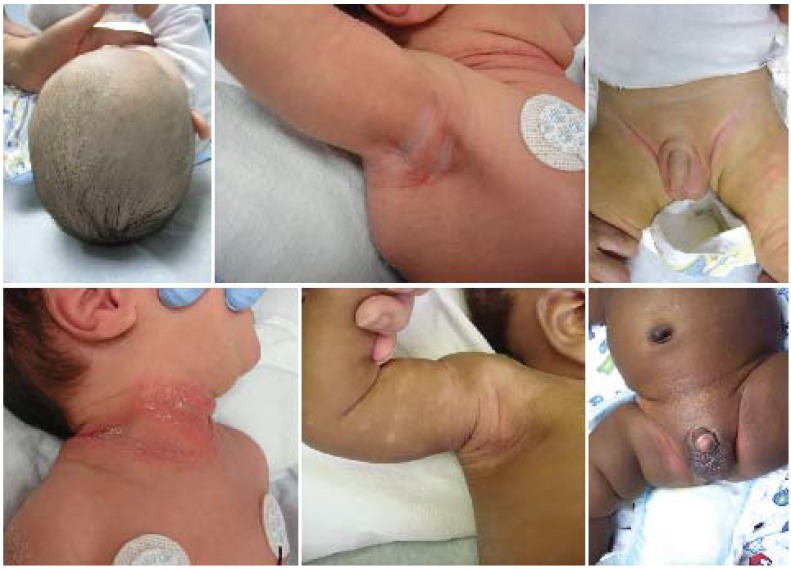
Seborrheic dermatitis.

Both AD and SD are common in infants, and can occur together [6] frequently leading to confusion. SD is distinguished by a lack of excoriations and sleep impairment. In infants, SD typically involves the face, scalp, and posterior auricular, nuchal, axillary, and/or inguinal folds. SD in adolescents and adults characteristically involves the scalp as well as alar and mesiolabial folds. SD lesions most often appear as salmon-pink patches with thick or greasy, white, off-white, or yellow scales (Figure 2). Lesions are many times hypopigmented, especially in patients with darker skin types, and may be misinterpreted as vitiligo.

SD affects male and female patients equally. Unlike AD, SD in infants usually resolves before two years of age. SD may be more prevalent in adults with neurologic or psychiatric disease. Associated alopecia is rare, so tinea capitis (see Section 3.3.1) should be considered in children with both hair loss and scalp lesions. The differential diagnosis of thick, tenacious scalp scale with or without associated alopecia includes psoriasis (see Section 2.2). Acute worsening with associated tenderness is characteristic of superimposed streptococcal intertrigo (see Section 3.1).

The cause of SD is not completely understood, but it is often associated with Malassezia species (previously Pityrosporum), because this organism heavily colonizes sebum-rich skin [7]. For this reason, topical anti-yeast treatment is often effectively used for long-term control [8], while TCS and TCIs may be used to quickly minimize redness and scaling [9].

### 2.2. Psoriasis

**Figure 3:**
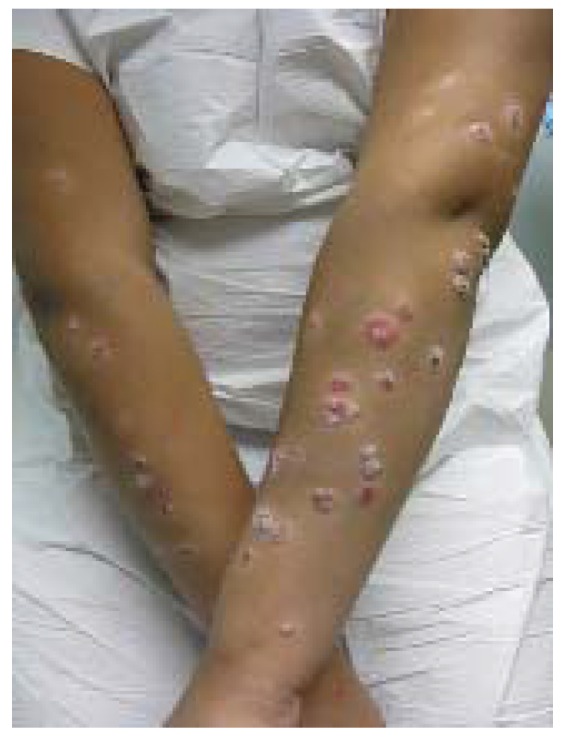
Psoriasis.

Psoriasis can affect patients of any age with no apparent gender bias. The peak age of onset appears to be adolescence/early adulthood [10,11], although psoriasis may be under-recognized in infants and children. Psoriasis vulgaris, or plaque psoriasis, is the most common form of psoriasis and is characterized by sharply circumscribed, persistent erythematous, indurated plaques with silvery-white, adherent scale and a predilection for extensor surfaces of the elbows or knees, or for the thumb in infants who suck their thumb (“thumb sign”) via the Köebner phenomenon (localization to areas of micro-trauma; Figure 3). However, other presentations are not uncommon and include acute widespread guttate, flexural (“inverse”), annular, palmoplantar, pustular, and scalp (pityriasis amiantacea, often accompanied by alopecia) psoriasis as well as psoriasis-eczema overlap (see Section 2.7) [12].

In infants and children, psoriasis may be misdiagnosed as AD because scaling is often less prominent and the distribution of lesions more often includes the face. Unlike AD, psoriasis is frequently encountered in the diaper area. Nail involvement (fine pits) is a subtle feature that can help differentiate psoriasis or coexisting psoriasis from AD in infants and children. Family history is often used as supporting evidence for diagnosis; however, only about one-third of patients have a family history of psoriasis, which suggests that it may be underdiagnosed.

Although Streptococcal pharyngitis or pharyngeal colonization is a well-recognized psoriasis trigger, impetigo, or other skin infections are rare [11].

TCS are often used as first-line treatment [12], but long-term TCS monotherapy can be associated with suboptimal response and rebound. In these cases, addition of a topical steroid-sparing medication (e.g.*,* vitamin D analog [13], salicylic acid [14] or TCI [15]) may improve efficacy and increase the chances of remission.

### 2.3. Nummular Dermatitis

Nummular dermatitis (ND) is characterized by round or oval (*i.e.*, nummular), well-demarcated lesions that are sometimes itchy. Lesions are usually asymmetrically distributed on the limbs, rarely on the face, but the rash may affect any area. Unlike AD, ND is unusual before age 5 [16].

The cause of ND is unknown, but because many cases are associated with localized microtrauma (e.g., insect bite, scratching), some consider this condition to be a form of psoriasis. Other suspected factors include *Staphylococcus aureus* colonization, contact allergens or irritants, xerosis, or stasis dermatitis.

### 2.4. Contact Dermatitis

Contact dermatitis (CD) is the most common form of dermatitis and like all dermatitis, acute CD is characterized by cutaneous erythema and edema. CD can be acute, chronic, persistent, or relapsing and it has been classically categorized as either irritant or allergic, but both types can coexist. In infants and children, allergic CD often occurs in the same distribution as irritant CD and may be underappreciated.

**Figure 4:**
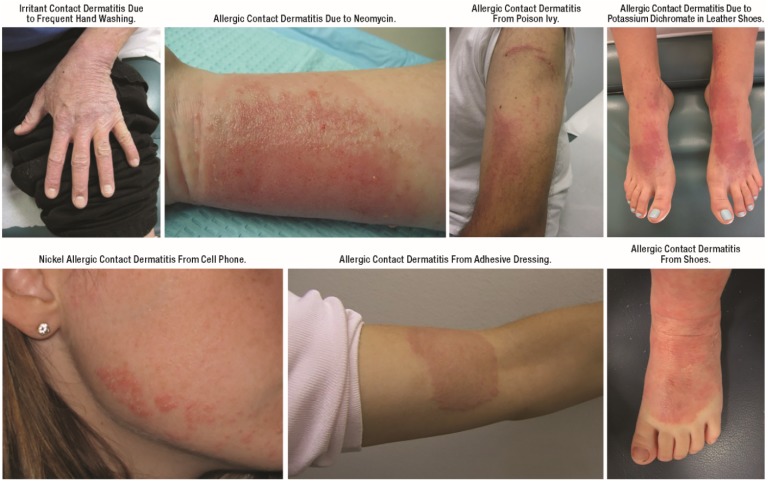
Contact dermatitis.

Allergic CD is a delayed-type hypersensitivity reaction, with onset after several hours to days after allergen exposure. The distribution is classically geographic and often asymmetric. Irritant CD is a more rapid response to physical barrier microtrauma, typically occurring within minutes, and is often less itchy, less geographic, and more symmetric (except in instances when allergen exposure is bilateral such as footwear or gloves) than allergic CD. Recognition of isolated CD relies on temporal pattern, as well as suggestive distribution (Figure 4).

CD commonly complicates other skin disease, including AD. Impaired skin barrier function has been a suspected risk factor but results of studies have been conflicting and risk may be allergen-specific [17,18,19,20]. Recognition of CD in the setting of coexisting skin disease requires a high index of suspicion based on personal/family history, presence of xerosis, and, if necessary, results of “patch testing” (although the sensitivity of patch testing is less than 70%). Diagnostic skill is also important for recognition of a less common manifestation of allergic CD called autoeczematization or “id” reaction, characterized by appearance of widespread, symmetrically distributed ectopic lesions [21,22].

Older adults have both greater lifetime risk of repeated allergen exposure and reduced skin barrier repair mechanisms, and so may be especially prone to allergic CD [23]. However, CD can affect patients of any age including infants and children, contrary to what had been previously believed [21,24]. In pediatric patients, irritant CD is most common on the face, dorsal aspect of the hands, and “diaper area”, often triggered by frequent cycles of skin wetting and drying as well as exposure to endogenous (e.g., drool, lip-licking, urine, and feces) or exogenous (e.g., cleansing products, highly alkaline or acidic foods) irritants. In adults, irritant and allergic CD most often presents as “hand dermatitis” triggered by frequent hand washing in healthcare and food industry workers. Common topical allergens include emulsifiers, preservatives, and fragrances used in topical products (e.g., diaper wipes, hand sanitizers, emollient lotions, and creams). Recognition of these triggers may be especially difficult when the allergen is an ingredient contained in products that have been used as a treatment for dermatitis. In some cases, sensitization with one allergen contained in a complex topical product will enable cosensitization to another ingredient in the same product. Nickel is another common sensitizer. Nickel allergic CD is classically localized to the infraumbilical area (from jean snaps) or ear lobules. Ear piecing in infancy is an important risk factor for nickel allergy.

The most effective way to alleviate CD is with strict avoidance of likely triggers. When triggers cannot be identified/avoided or there is residual dermatitis after triggers have been removed, TCS [25,26] or TCIs [27,28,29] may reduce inflammation; however, chronic use of TCS should be avoided, especially on the face and diaper area. Long term and frequent (greater than once daily) application of TCS (especially higher potency preparations) is contraindicated, due to an increased risk of skin barrier compromise and systemic exposure.

### 2.5. Dermatographism

**Figure 5:**
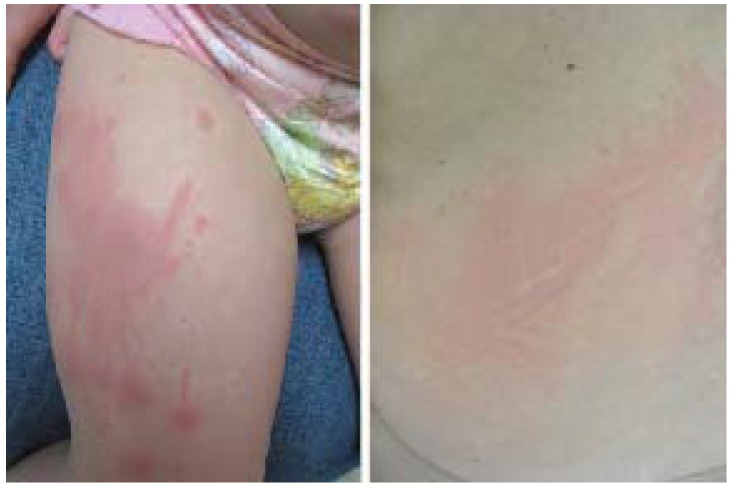
Dermatographism.

**Figure 6:**
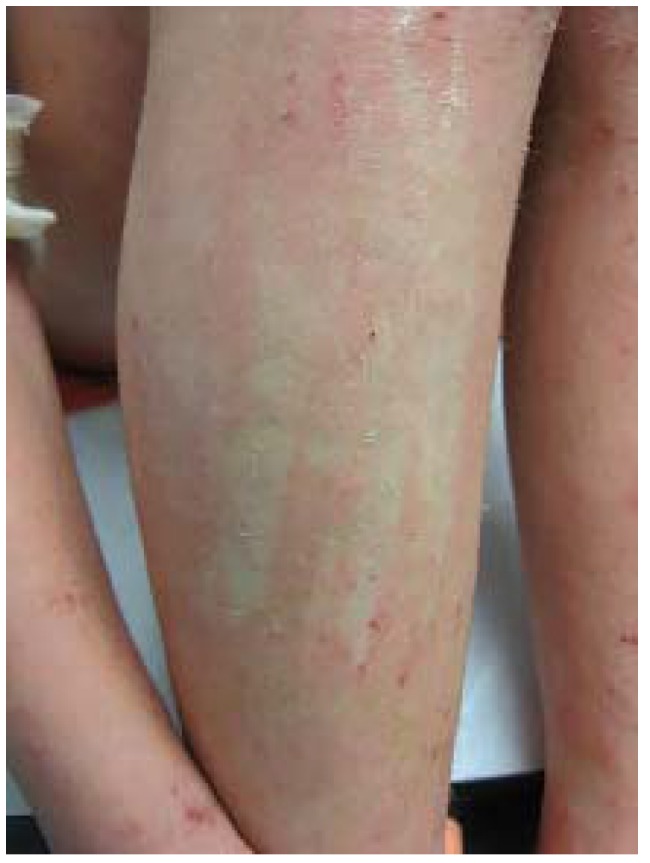
White dermatographism (typically observed in atopic dermatitis).

Dermatographism is an immunologic response to pressure applied the skin, characterized by local wheal-and-flare erythema followed by edema and itch (Figure 5). Alternately known as dermographism, dermatographic urticaria, or mechanical urticaria, signs and itch classically begin within 5 min of stimulation and persist for 15–30 min. Delayed-type dermatographism is much less common, presenting as an urticarial response 3–6 h after stimulation, and lasting for 24–48 h, not always preceded by an immediate wheal-and-flare. “White dermatographism” is a minor diagnostic criterion for AD and presents as pallor rather than erythema, with subsequent erythematous halo (Figure 6).

Patients of any age can be affected by dermatographism but it is most often diagnosed in young adults. Onset is generally gradual and resolution is often within five years [30]. Dermatographism can affect any region of the body including the palms and soles, but not usually the scalp or genitals. Tight clothing is a common trigger. Diagnosis is clinical and confirmed by the appearance of a wheal in response to mechanical stimulation. Although dermatographism is usually idiopathic, in some cases it is associated with drug reactions, scabies, stress/anxiety, or an underlying systemic disease (e.g., hyperthyroidism, type 2 diabetes mellitus). Trigger avoidance is the most effective way to control dermatographism.

### 2.6. Pityriasis Alba

Pityriasis alba (PA) is a common, idiopathic, generally asymptomatic condition, often noted as an incidental finding on skin exam. PA is associated with AD, as a minor diagnostic criterion. PA is most often found in school-age children with no gender bias [31].

**Figure 7:**
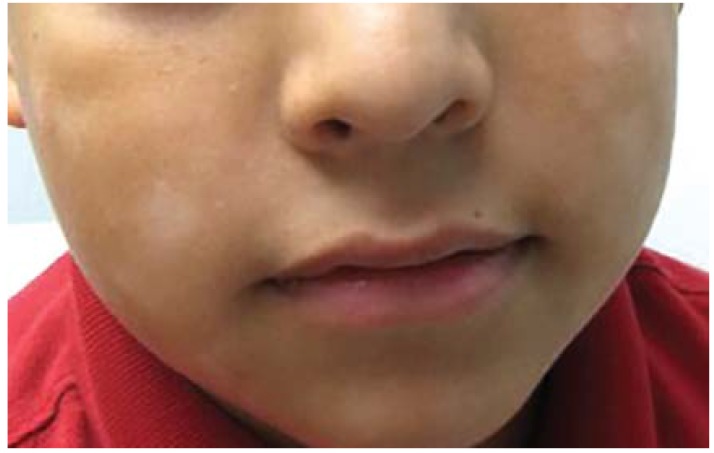
Pityriasis alba.

PA appears as subtle, poorly circumscribed, hypopigmented patches with fine papular follicular accentuation, most often on the proximal upper extremities, although the face and trunk are sometimes involved (Figure 7). Patches become more prominent with increased summer sun exposure, as surrounding skin tans. In winter, hypopigmentation is less prominent, and the patches feature powdery, white scale. Some reports have suggested that PA is more common in patients with darker skin, but the condition is equiprevalent in all skin types, just more apparent on darker skin tones [31]. Severe cases of PA are clinically indistinguishable from hypopigmented mycosis fungoides (see Section 5.2).

Emollients can minimize scaling but will not impact hypopigmentation, which may persist for months to years despite control of xerosis and inflammation. A few reports have documented efficacy of TCIs [32,33].

### 2.7. Overlap

**Figure 8:**
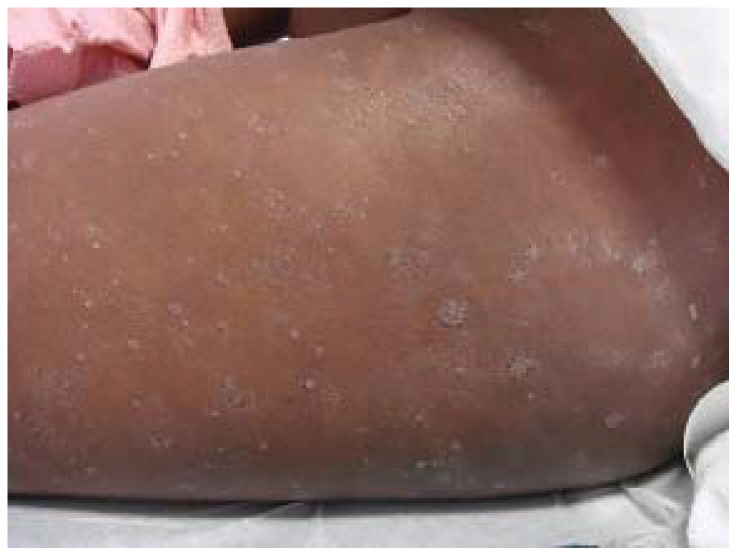
Psoriasis-eczema overlap.

“Overlap” is a term used to describe one or more coexisting inflammatory skin diseases. The most well described combination may be psoriasis-eczema overlap (PsE), also known as eczematous psoriasis and PsEma. Patients with PsE typically present with a combination of flexural eczema and psoriatic lesions that lack thick plaques and are more likely to experience itch than patients with isolated psoriasis (Figure 8) [12,34,35]. In one study, PsE responded well to psoriasis treatment strategies including TCS [34].

## 3. Infections

### 3.1. Bacterial Infections

#### 3.1.1. Impetigo

**Figure 9:**
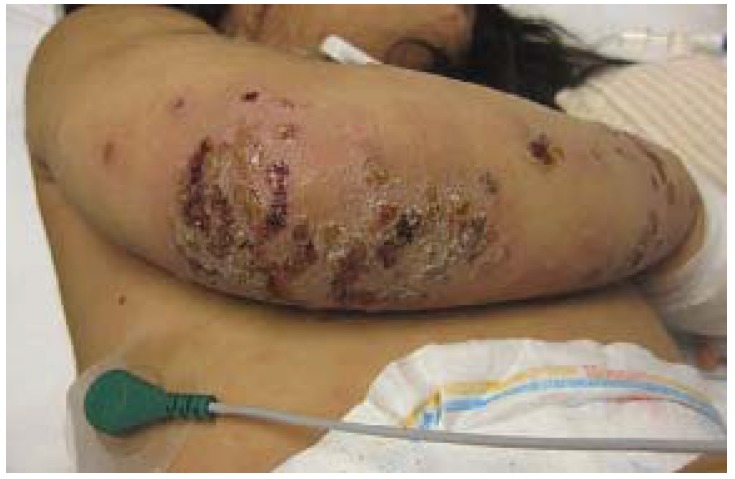
Impetigo.

Impetigo is a superficial, cutaneous bacterial infection that most often occurs at sites of minor skin trauma (e.g., traumatic injury, irritation, dermatitis, insect bites). Impetigo is most often caused by *S. aureus* or *Streptococcus pyogenes* and is characterized by erythema, edema, and tenderness, often with honey-yellow crusting (Figure 9). Less common variants include bullous impetigo and Streptococcal intertrigo, which are classically superimposed on SD (see Section 2.1). Pharyngeal or perianal *Streptococcus* carriage is a risk factor for Streptococcal impetigo. Impetigo is most common in young children, but can occur at any age, with a higher incidence in males [36].

Both Staphylococcal and Streptococcal impetigo are easily treated with skin cleansing and topical antibiotics. Dilute bleach baths may also be helpful and will not contribute to the risk of microbial resistance. Oral antibiotics are indicated for widespread disease, or accompanying fever. Patients with recurrent impetigo may benefit from skin surface, nares, pharyngeal, or perianal cultures for mucosal carriage detection, bacterial identification, and antimicrobial resistance determination.

Patients with AD are heavily colonized with *S. aureus*, on both dermatitic and normal-appearing skin, so skin cultures cannot differentiate colonization from true infection. True impetiginization in AD is most often characterized by acute onset with skin tenderness and crops of pustules. Despite frequent use of antibiotics among patients with AD, a high incidence of methicillin-resistant *S. aureus* (MRSA) has not been found in this population [37,38,39]. Mupirocin resistance is emerging [40]. Control of impetiginized AD may be initially managed with dilute bleach baths.

#### 3.1.2. Secondary Syphilis

**Figure 10:**
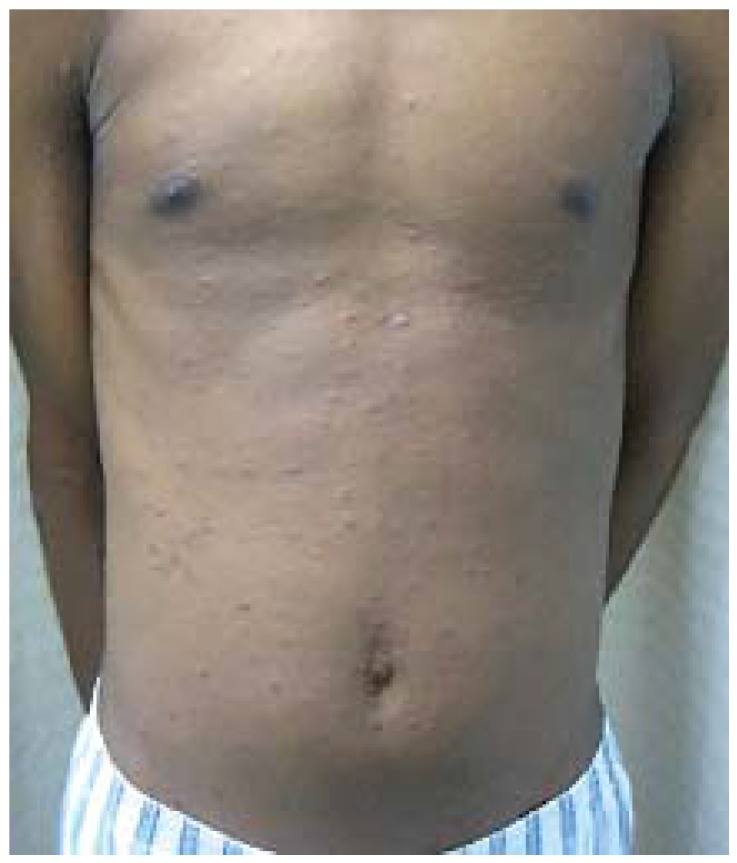
Secondary syphilis.

**Figure 11:**
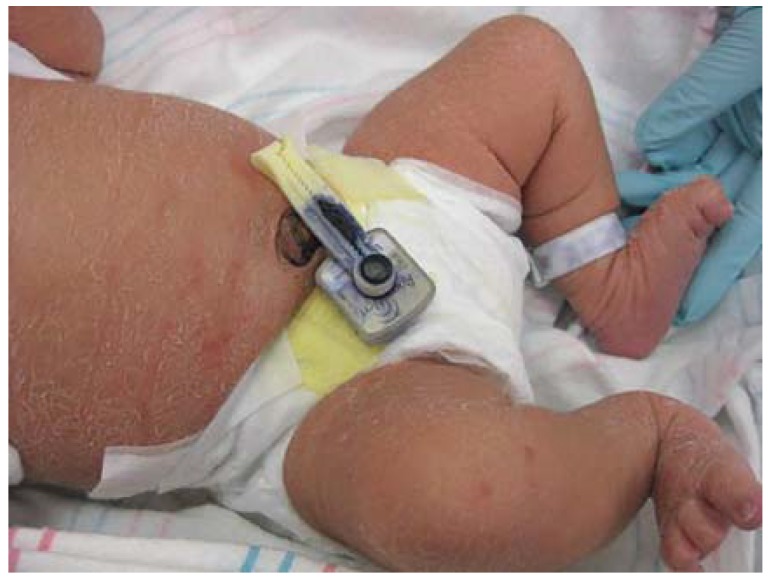
Congenital syphilis.

Widespread availability of antibiotics has made secondary syphilis (SS) a rare condition in all but immunocompromised patients; however, this condition, historically known as “the great mimicker”, is easily cured and should not be overlooked. In contrast to the local reaction observed in primary syphilis (often a single chancre), SS classically presents as a widespread eruption several weeks to months after appearance of the primary chancre (Figure 10) [41]. Most syphilitic disease in children is congenital and thus secondary due to the hematogenous spread from mother to fetus *in utero* (Figure 11).

Secondary syphilis can manifest in a number of ways but most often as a diffuse macular eruption that evolves into maculopapular or pustular lesions on the trunk and proximal extremities; however, any skin surface can be involved including the palms and soles [42]. Scaling, mucosal ulceration, patchy hair loss, and condyloma latum (greyish-white moist raised patches found on moist skin surfaces) may also be present. SS is not associated with pruritus. Cutaneous symptoms are often accompanied by fever, lymphadenopathy, malaise, weight loss, and headache.

### 3.2. Viral Infections

#### 3.2.1. Molluscum Dermatitis

**Figure 12:**
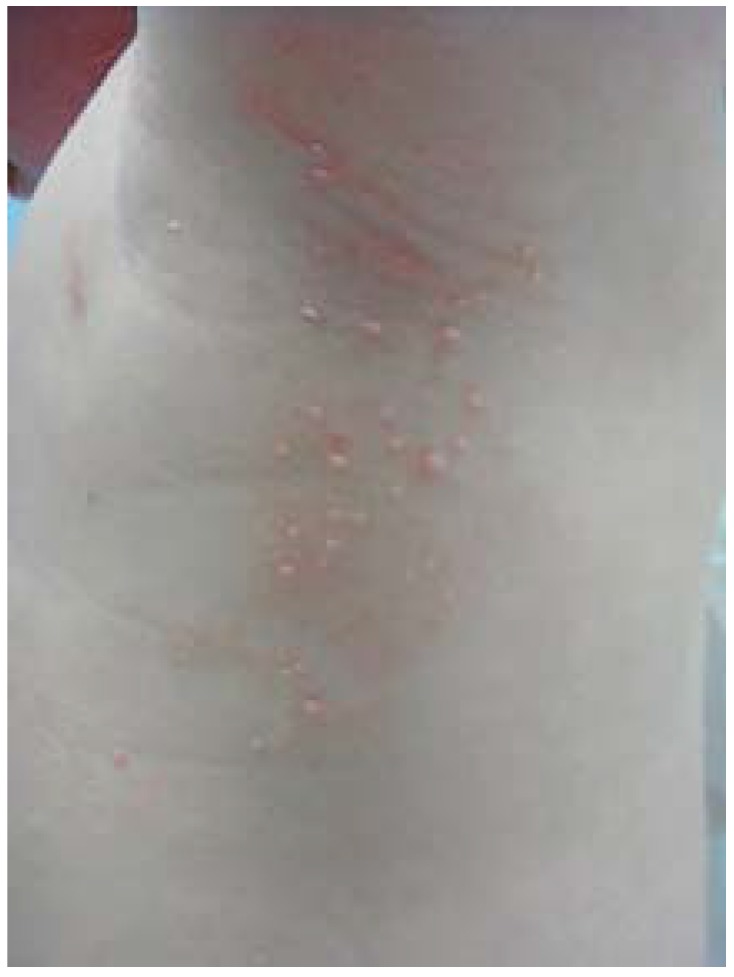
Molluscum dermatitis.

In most patients, molluscum contagiosum (MC) causes a relatively benign infection characterized by scattered clusters of small, umbilicated, flesh-colored, pink, or “pearly” white papules. For some patients, diffuse or discoid dermatitis develops surrounding some of these MC papules (molluscum dermatitis; MD) often masking the papules altogether (Figure 12). Patients (with or without AD) that develop MD have a prolonged and/or more severe course [43,44] possibly due scratching and autoinoculation to other skins sites; however, in some patients the apparent “spread” of MC lesions may be due to autoeczematization [45]. Secondary infection is uncommon and characterized by acute onset of tenderness and drainage.

Children are primarily affected with no apparent gender bias. Approximately one-quarter of patients with MC develop MD [44,46] while half of patients with AD do so [44]. There is some speculation that patients with AD may also be more susceptible to MC, but data are not conclusive [44,46,47,48].

Uncomplicated MC does not usually require treatment; however, for MD, short-course TCS may help control the dermatitis and prevent spread [44]. Data are conflicting as to the effect of TCS on recurrence rate [44,46].

#### 3.2.2. Eczema Herpeticum

**Figure 13:**
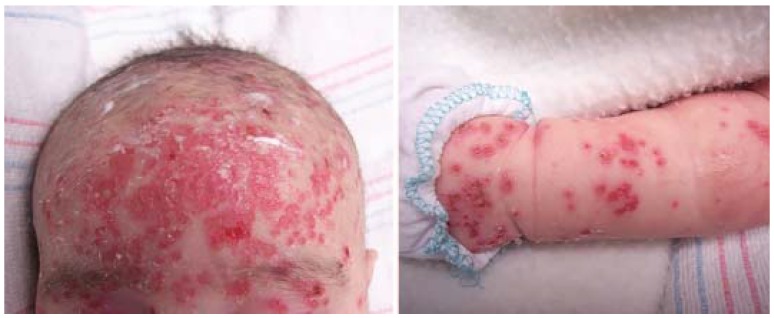
Eczema herpeticum.

Eczema herpeticum (EH), is an acute-onset, potentially life-threatening viral infection caused by herpes simplex virus occurring almost exclusively in patients with a history of chronic skin disease, especially AD. Patients present with widespread tender “punched out” erosions with a predilection for the face and areas of chronic dermatitis (Figure 13). Regional lymphadenopathy is often present.

EH may be easily mistaken for impetigo (see Section 3.3.1.), especially for recurrent EH in patients with severe AD (“EH incognito”). Positive skin surface bacterial culture for with Staphylococcal or Streptococcal bacteria is common, and does not exclude EH. Diagnosis can be confirmed by skin scraping for Tzanck smear, viral culture, PCR, or immunofluorescence, but sensitivity is low for all these techniques, so a high index of suspicion is important. Empiric antiviral treatment is often indicated; however, acyclovir resistance is emerging [49]. Continued monitoring after clearance is recommended because EH recurs in about half of patients [50].

EH can affect patients of any age, but it is most common in infants and children. Approximately 10%–20% of patients with AD develop EH and those that do typically have more severe/earlier onset AD, higher prevalence of atopic comorbidities, and are more likely to have a history of *S. aureus* or MC infection [50,51]. Previous TCS use has been cited as a possible risk factor for EH [52]. Thus far, data are not sufficient to determine the effect of TCIs on EH risk [52,53,54].

#### 3.2.3. Eczema Vaccinatum

Kaposi’s varicelliform eruption was used historically to refer to eczema vaccinatum (EV) or EH, conditions with similar clinical features and risk factors. EV is a rare, potentially life-threatening eruption that develops in predisposed individuals either after immunization for smallpox with live, attenuated vaccinia virus or physical contact with a recently vaccinated individual [48]. Although universal smallpox vaccination successfully eradicated the disease in the 1970s, recent bioterrorism concerns have prompted a reinstitution of smallpox vaccination for military personnel leading to a few recent reports of EV.

Risk factors include a history of atopy (even in the absence of coexisting dermatitis), or other primary skin disease featuring barrier dysfunction (e.g., dermatitis herpetiformis (DH; see Section 8.4) or primary immunodeficiency disorders (see Section 7.3)). EV presents as a rapidly progressing generalized, vesiculopustular, smallpox-like eruption appearing first and more densely in areas of cutaneous compromise.

#### 3.2.4. Eczema Coxsackium

Initially reported in 2013, a new variant of Kaposi’s varicelliform eruption (eczema coxsackium (EC)), attributable to coxsackievirus A6 (CSVA6), is being increasingly recognized. EC is related to the well-described hand, foot, and mouth disease (HFMD), which is most often caused by CSVA16. Over half of CSVA6-related HFMD manifests as EC [55].

**Figure 14:**
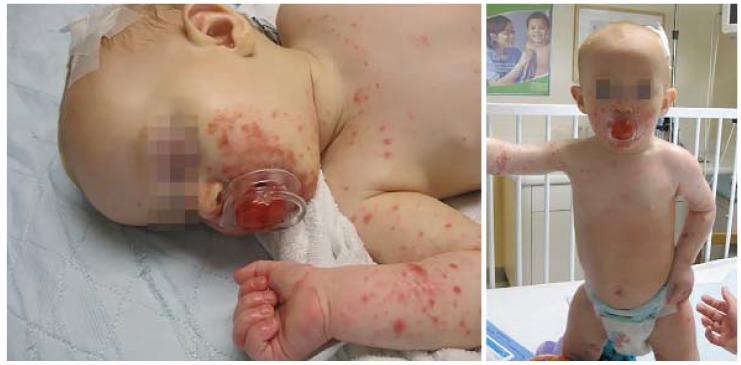
Eczema coxsackium.

In contrast to the oral erosions and gray-white, oval vesicles on the hands, feet, and buttocks typically associated with HFMD, EC manifests as EH-like lesions with a predilection for hemorrhagic vesicles within dermatitic skin (Figure 14). While the cutaneous findings of EC are the most dramatic presenting sign, children with AD typically have associated fever and constitutional symptoms as well as subsequent onychomadesis.

A majority of patients with EC have a history skin disease, most often AD, and patients with a history of AD are significantly more likely to manifest EC [55]. A diagnosis of EC is most reliably confirmed by serum PCR for coxsackievirus. Treatment is supportive, with antipyretics and bland skin care.

#### 3.2.5. Viral Exanthem

**Figure 15:**
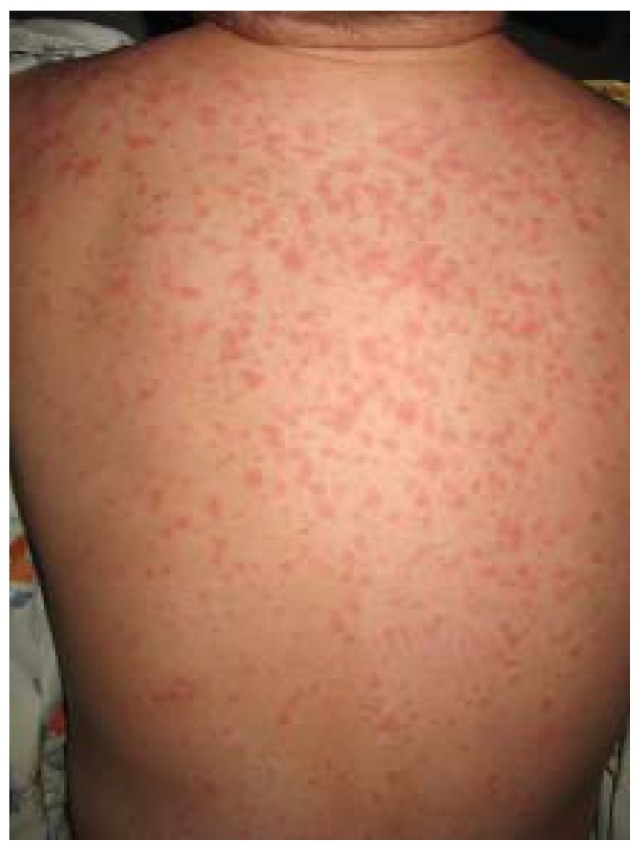
Viral exanthem.

Viral exanthems (VE) are a heterogeneous group of skin findings associated with a wide variety of systemic viral infections. The most common VE is acute onset of generalized morbilliform eruption, featuring widespread, fine, pink macules and/or papules that may be confused with AD (Figure 15). Other VEs include more pathognomonic features: unilateral laterothoracic exanthem (asymmetric periflexural exanthem of childhood), purpuric socks and gloves syndrome, infantile popular acrodermatitis (Gianotti-Crosti syndrome), and fifth disease [56]. VEs are generally self-limited and treatment is supportive.

### 3.3. Fungal Infections

#### 3.3.1. Tinea

**Figure 16:**
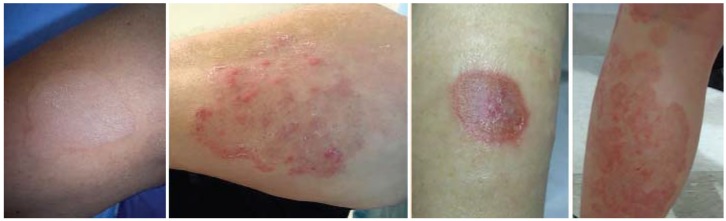
Tinea corporis.

**Figure 17:**
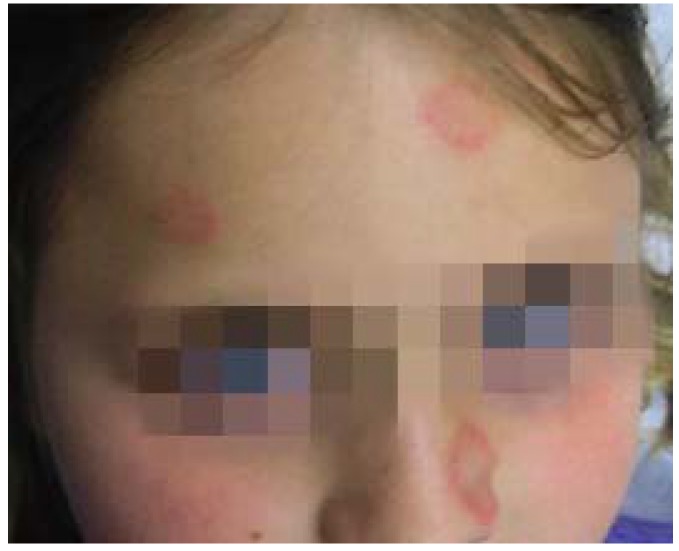
Tinea faciei.

Tinea, or “ring worm”, is a dermatophyte infection of the skin that characteristically appears as well demarcated, annular, red, scaly, usually pruritic patches with central clearing and advancing border (Figure 16 and Figure 17). Infections may be acute with sudden onset and rapid spread or chronic with a slow extension of mild rash.

**Figure 18:**
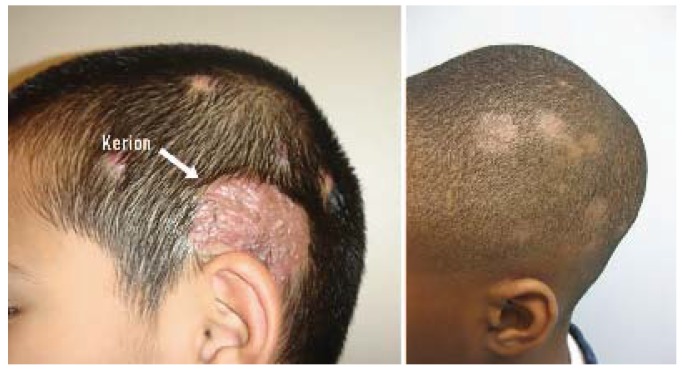
Tinea capitis.

Infants and children most often present with tinea capitis [57]. Tinea capitis can vary in presentation from pruritus and scales with patches of alopecia, broken hairs, and crusting on the scalp to tender boggy plaques and pustules (kerion) (Figure 18). In many cases, cervical or suboccipital lymphadenopathy is also present. Prevalence is greatest among African Americans and among children ages 3–9 [58]. Children presenting with tinea corporis or faciei often harbor occult tinea capitis. Definitive tinea capitis treatment requires systemic antifungal for 4–6 weeks.

Data do not support an increase susceptibility to tinea among patients with AD; however, the broken skin, erosions, and excoriations associated with AD are subject to tinea fungal infections. Tinea misdiagnosed as AD may improve but not clear after application of TCS, a condition known as “tinea incognito”.

#### 3.3.2. Candidiasis

**Figure 19:**
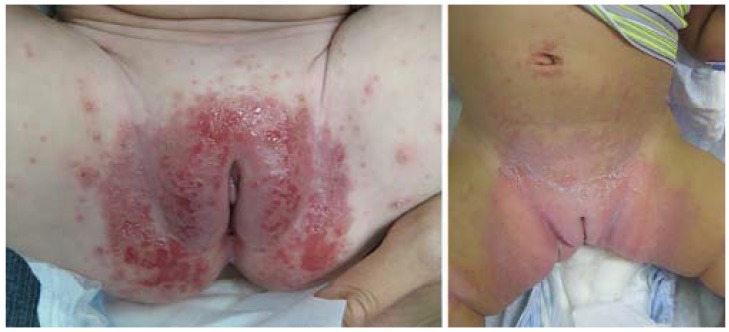
Candida culture-positive diaper dermatitis.

Although irritant CD is the most common cause of diaper rash, Candida is a frequent cofactor (Figure 19). Widespread cutaneous Candida infection is uncommon, occurring most often in infants. In adolescents and adults, cutaneous Candidiasis most often localizes to skin folds, especially in individuals with predisposing factors such as obesity, diabetes, and immunosuppression. Infantile candidiasis has been categorized into two subsets: congenital and neonatal. Congenital candidiasis presents at birth with generalized erythroderma and “burn-like” desquamation that may obscure the diagnosis. At risk infants are premature, low both weight, and born to mothers heavily colonized with Candida, often treated with antenatal antibiotics. Congenital candidiasis is associated with significant morbidity and mortality. Neonatal Candidiasis presents in the first 1–2 weeks of life with patchy redness and scaling in an otherwise healthy, generally full-term infant with proximal nail dystrophy and paronychia as a subtle associated finding.

## 4. Infestations

### 4.1. Scabies

**Figure 20:**
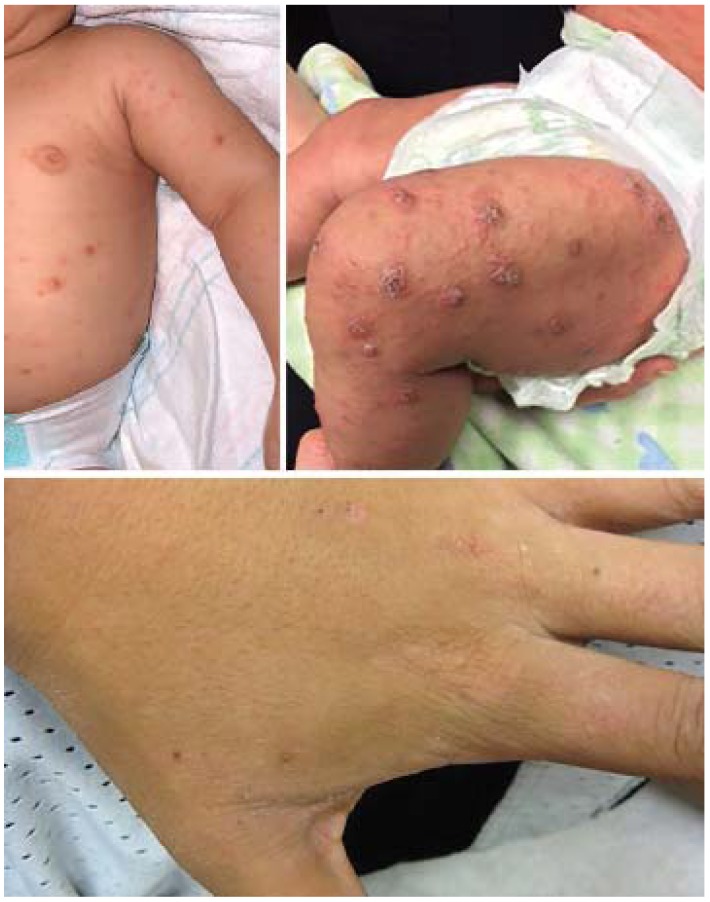
Scabies.

Scabies is an allergic reaction to the eggs and feces of the female *Sarcoptes scabei*. The reaction is characterized by small, red papulovesicles or dermatitic lesions. In infants, the most commonly affected areas are palms, soles, face, and scalp. In adults, finger webs, wrists, areolar area, and genitals are most often affected (Figure 20). Patients frequently report an insidious onset of pruritus that is especially intense at night, although not all patients will experience itch.

**Figure 21:**
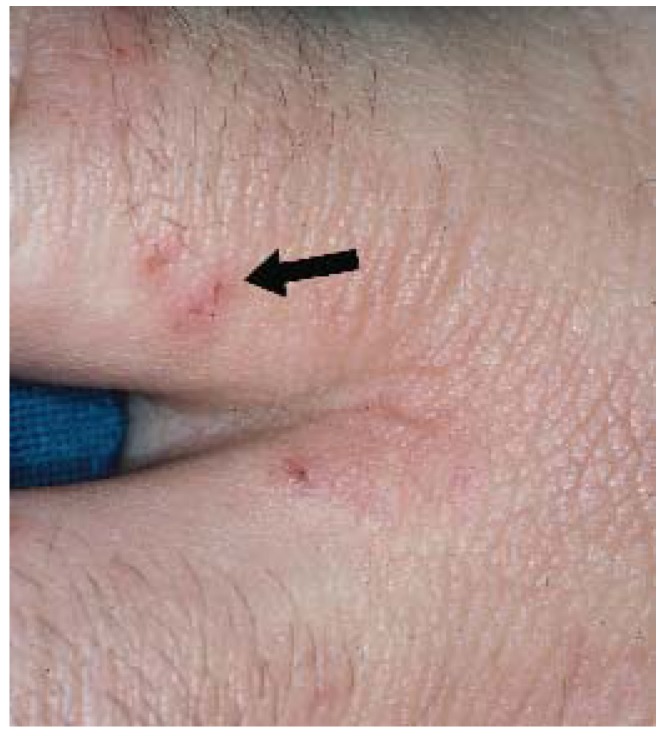
Scabies infestation with visible burrow.

Scabies is most often diagnosed in young children and young adults [59], but it is not limited to these age groups or to a particular socioeconomic group. The presence of gradual onset of itch, lack of xerosis, and other family members being itchy with similar time of onset helps to confirm diagnosis. Visualization of the mite’s linear burrows (tiny grey irregular tracks) or positive scrapings for mites, eggs, or scybala is definitive (Figure 21).

Patients with AD are more prone to and have more severe reactions to the scabies mite. Treatment with TCS may improve appearance (“scabies incognito”), but prolong the infestation.

## 5. Malignancies

### 5.1. Letterer-Siwe Disease

Letterer-Siwe Disease (LSD) is a malignant form of Langerhans cell histiocytosis (LCH) and is the type of LCH most likely to be mistaken for AD. LCH classically presents in infants with crusted, scaly, SD-like dermatitis or ulcerations on the scalp (most often), periauricular, perineal, and/or axillary regions. Reddish-brown purpuric pustules (petechiae) may also be present.

### 5.2. Cutaneous T-Cell Lymphoma

Cutaneous T-cell lymphoma (CTCL) onset before the age of 50 is rare, but an increasing incidence in the pediatric population has been reported over the past 10 years [60]. The early stages of CTCL present as slowly-progressing scaly patches/plaques (mycosis fungoides) or quickly-progressing generalized erythema (Sézary syndrome) on the trunk. Tumors and pruritus are generally present at later stages. In children, CTCL most often presents with wide-spread poorly circumscribed hypopigmented macules (hypopigmented mycosis fungoides), indistinguishable from severe PA (see Section 2.6). Lesions may respond favorably to TCS, delaying diagnosis. There is evidence that severe AD may be a risk factor for CTCL [61,62]. Lack of atopy, adult onset, and symptoms accompanied by weight loss or malaise are key to differential diagnosis.

## 6. Genetic Disorders

### 6.1. Keratosis Pilaris

**Figure 22:**
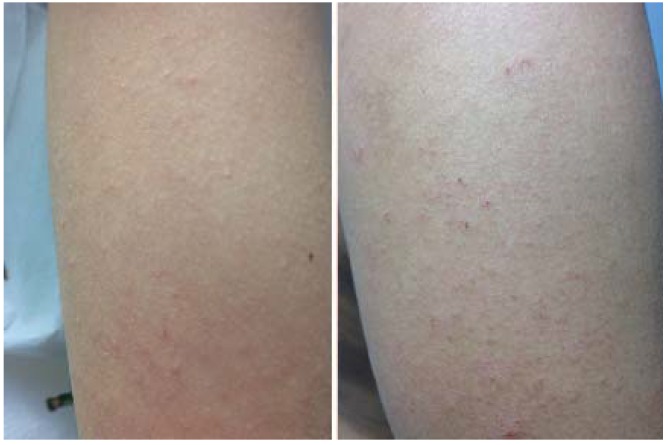
Keratosis pilaris.

Keratosis pilaris (KP) is a benign, generally asymptomatic disorder of follicular hyperkeratinization causing small, rough papules associated with varying degrees of erythema and sometimes-mild pruritus (Figure 22). This characteristic stippled or “gooseflesh” appearance is usually observed with dry scaly skin on the cheeks or extensor surfaces of the upper arms, upper legs, and buttocks. KP is often associated with AD or ichthyosis vulgaris (IV; see Section 6.2).

TCIs (tacrolimus specifically) have been effective in reducing erythema [63]. Emollients containing urea, salicylic acid, lactic acid, or glycolic acid may temporarily improve the appearance of the papules (but worsen the erythema).

### 6.2. Ichthyoses

Ichthyoses are a group of congenital diseases characterized by universal scaling, among which IV is the most common [64].

#### 6.2.1. Ichthyosis Vulgaris

**Figure 23:**
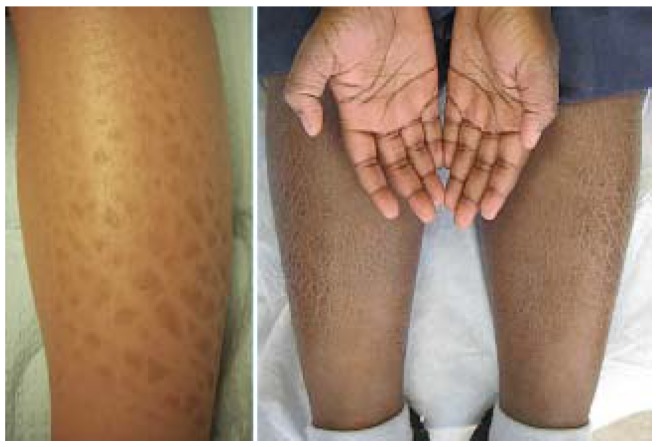
Ichthyosis vulgaris (**left**) and associated palmar hyperlinearity (**right**).

IV is characterized by xerosis and fine, or sometimes coarse, light grey, centrally adherent scale that usually appears 2–6 months after birth, increases though puberty, and decreases with age thereafter. Changes are accentuated on the legs where scales may take on a mosaic appearance (Figure 23). The palms and soles of patients with IV are characteristically, but not always, hyperlinear.

Approximately half of patients with IV develop AD [64] and the xerosis observed with AD may be difficult to differentiate from IV. IV is associated with earlier onset and severity of AD as well as atopy [65]. IV is also associated with KP (see Section 6.1). Unlike AD, few patients experience pruritus or skin inflammation.

IV can be distinguished from most other forms of ichthyosis by sparing of antecubital and popliteal fossae.

#### 6.2.2. Other Ichthyoses

Infantile presentation of other ichthyoses is virtually identical, so distinguishing between them is nearly impossible until after the first year of life, when distinctive features begin to manifest. X-linked recessive ichthyosis (XLRI), a recessive ichthyosis found overwhelmingly in males, is characterized by erythema and generalized scaling at or within weeks of birth. The large, translucent, peeling scales present early give way to adherent, light brown or grey, rhomboid scales as the patient ages. Scaling is most prominent on the extensor surfaces, neck, face, trunk, and buttocks. Palms and soles are not affected.

Babies with autosomal recessive congenital ichthyosis (ARCI) are generally born with or develop soon after birth a thickened collodion-like sheet that encases the entire body (*i.e.*, “collodion baby”). After approximately one month, this “sheet” is replaced by varying degrees of generalized scaling in a majority of patients. In lamellar ichthyosis, a form of ARCI, scaling is large, dark brown, plate-like scaling (without erythema) that is most prominent on flexural surfaces, the forehead, and lower limbs. The nonbullous ichthyosiform erythroderma form of ARCI is characterized by fine, white scales with generalized erythroderma (but on the legs scaling may be darker, larger, and plate-like). Pruritus is a common feature of ARCI.

## 7. Immunodeficiency Disorders

### 7.1. Netherton Syndrome

Netherton syndrome (NS) is a rare condition that generally presents shortly after birth with severe erythema and scaling typically on the scalp, face, and eyebrows. In some cases, patients develop a pruritic eczematous rash that is unresponsive to treatment. At 1–2 years of age, the erythroderma may be replaced by an annular and polycyclic pattern of lesions (ichthyosis linearis circumflexa), especially in girls.

Severe skin inflammation and related infections may be life threatening. Dehydration and failure thrive are also typically present. The presence of “bamboo hair” (trichorrhexis invaginata; short, brittle, lusterless hair) is diagnostic.

TCS are not recommended for NS due to a high risk of systemic exposure. Systemic exposure is also high for topical tacrolimus (a TCI) [66]. Systemic exposure for topical pimecrolimus (another TCI) was detectible, but lower than expected and pimecrolimus treatment has been effective and well tolerated in NS [67].

### 7.2. HIV/AIDS-Related Skin Changes

As of 2011, 57 different cutaneous skin disorders have been linked to HIV/AIDS and nearly every patient with HIV/AIDS will experience some cutaneous changes, many of which mimic AD [68]. These HIV/AIDS-related cutaneous changes are more often than not secondary to immunosuppression (*i.e.*, opportunistic infections or increased susceptibility to malignancy); however, a significant number of inflammatory dermatoses are directly related to HIV infection. The mechanisms by which HIV infection effects these changes has not been completely elucidated but there is evidence to suggest that they may be related to changes in sweat/oil production, depletion of Langerhans cells, decreased CD4+ T cell function, and/or shifts in cytokine profile.

SD (see Section 2.1), psoriasis (see Section 2.2), eosinophilic folliculitis (erythematous urticarial vesiculopapular or pustular rash affecting the face, neck, and upper chest/back), primary HIV viremia (morbilliform eruption affecting the trunk and limbs), pruritic papular eruption of HIV (diffuse red rash affecting the trunk and face), xerosis, and AD have each been linked directly to HIV infection. AD is observed in ~30%–50% of HIV/AIDS patients, particularly AIDS patients [68]. Patients with HIV/AIDS also often report drug eruptions (DE; see Section 9.2) possibly related to HIV-related changes in immune function and/or metabolic dysfunction.

Treatment is often disappointing, as HIV-related cutaneous changes are particularly resistant to treatment and/or prone to recurrence. Highly active antiretroviral therapy has significantly reduced the incidence of opportunistic infections and Kaposi’s sarcoma, but it has had little effect on the incidence of inflammatory dermatoses. TCS have limited efficacy [68].

### 7.3. Other Immunodeficiency Disorders

STAT3 deficiency and DOCK 8 deficiency are rare phenotypes of primary immunodeficiency that feature elevated serum IgE, eosinophilia, susceptibility to cutaneous and sinopulmonary infections, predisposition for malignancy (predominantly lymphoma), and eczematous dermatitis [69,70,71]. The cutaneous symptoms associated with STAT3 deficiency usually begin as a pustular rash on the face or scalp at or just after birth, much earlier than AD. The rash may resolve or persist and evolve into eczematous dermatoses. On the other hand, patients with DOCK8 deficiency tend to develop eczematous dermatoses a few months after birth, much like AD. These cutaneous symptoms are often the first symptom of immunodeficiency. Treatment is mainly palliative and may be difficult as TCS or TCIs may exacerbate cutaneous infections.

Wiskott-Aldrich syndrome (WAS) is an rare X-linked recessive disorder characterized by elevated IgE, lymphopenia, susceptibility to infections, predisposition for malignancy (primarily hematopoietic), eczematous dermatitis, and/or bleeding due to thrombocytopenia and platelet dysfunction [69]. The first clinical sign of WAS is often bleeding-related, however eczematous dermatitis generally develops within the first few months of life [69] and is indistinguishable from AD including in its anatomical distribution. Much like AD, WAS-related dermatitis is pruritic and often improves with age. Patients are also prone to atopy. TCS have been effective in treating cutaneous symptoms [69].

Immunoglobulin (Ig) A deficiency (IgAD) and IgM deficiency are associated with eczematous dermatitis which is often mistaken for AD.

Leiner phenotype is a broad spectrum of immunodeficiency disorders (including severe combined immunodeficiency syndrome [SCID]) in infants characterized clinically by exfoliative dermatitis, chronic diarrhea, failure to thrive, and recurrent bacterial and Candida infections. These infants manifest noncongenital or acquired erythroderma within the first few weeks of life. Patients with SCID generally present in early infancy with recurrent mucocutaneous infections, erythroderma, and morbilliform or SD-like eruptions. Individuals may also present with cutaneous manifestations of graft-*versus*-host disease (GVHD; see Section 9.1) such as exfoliative dermatitis, a lichenoid rash, or sclerodermoid skin changes [72]. Without treatment, SCID is lethal. Cyclosporine can be used to treat dermatitis. There are nine described SCID subtypes, each caused by a different genetic mutation. Among these, patients with Omenn syndrome (OS) present with erythroderma, eosinophilia, failure to thrive, chronic diarrhea, lymphadenopathy, and hepatosplenomegaly. Symptoms usually manifest before the age of six months. Presence of lymphadenopathy and hepatosplenomegaly distinguish OS from other forms of SCID and may lead to it being confused with graft-*versus*-host disease (GVHD; see Section 9.1).

Hypohidrotic ectodermal dysplasia (HED) is an X-linked trait caused by hypomorphic mutation in the *IKBKG* (or *EDA*) gene, encoding nuclear factor κB essential modulator (NEMO). HED presents in males as persistent and extensive SD- or AD-like skin eruptions, intertrigo, and facial ectodermal dysplasia including absent or sparse hair growth, delayed tooth eruption, alopecia, dry wrinkled skin, and signs of immunodeficiency such as sepsis, pneumonia, otitis media, sinusitis, lymphadenitis, bronchiectasis, skin and soft tissue infections, and/or infections of the bones and gastrointestinal tract. Heterozygous females show milder symptoms, if any.

Autoimmune polyendocrinopathy-candidiasis-ectodermal dystrophy (APECED) syndrome is an extremely rare autosomal recessive genetic disorder. Patients with APECED initially present with recurrent mucosal and cutaneous Candidiasis typically before the age of 5 with no other opportunistic infections [69]. Subsequently, hyperparathyroidism and autoimmune adrenal insufficiency develop. Individuals may also have type 1A diabetes, hypogonadism, pernicious anemia, malabsorption, alopecia, and vitiligo.

In rare cases, patients with iatrogenic immune deficiency develop paradoxic inflammatory skin disease (PISD) with features of chronic eczema or psoriasis [73]. The most well described PISD is psoriasis triggered by anti-TNF therapy (infliximab, etanercept, adalimumab). Less well appreciated is an AD-like chronic skin disease observed in immunocompromised children treated with long-term systemic calcineurin inhibitors (cyclosporine, tacrolimus). Recognition of PISD requires a high index of suspicion and treatment is challenging.

## 8. Nutritional Disorders

**Figure 24:**
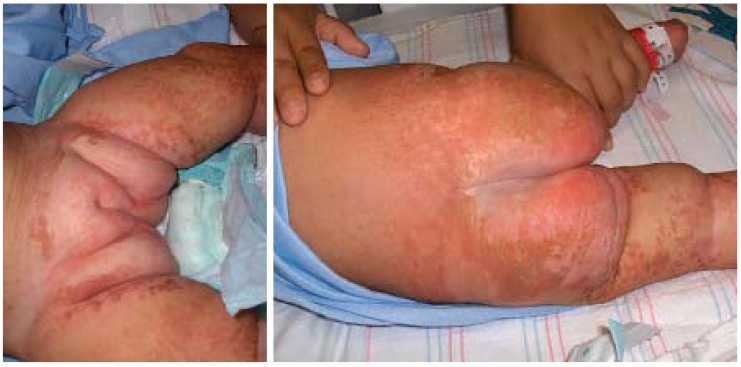
Kwashiorkor.

Cutaneous symptoms of nutritional disorders can be the result of poor nutrient intake/anorexia (Kwashiorkor (Figure 24), zinc deficiency (ZD)), malabsorption (dermatitis herpetiformis (EH), nutritional deficiency dermatitis of cystic fibrosis (CFNDD)), or impaired end-organ response (phenylketonuria (PKU), biotinidase deficiency (BD)).

### 8.1. Cystic Fibrosis

In infants, the initial symptoms of CF may be CFNDD. This potentially life threatening nutritional deficiency presents as erythematous annular papules that are distributed periorally, periocularly, or in the “diaper area”. The eruption then spreads to the flexural surfaces of the extremities and takes on a “peeling paint” scaly appearance. In addition, patients demonstrate sparse brittle hair, periorbital and extremity edema, and failure to thrive. CFNDD generally presents during the first weeks to months of infancy and precedes pulmonary and gastrointestinal symptoms by a few months. Though this is an extremely rare presentation, these manifestations are associated with false-negative sweat test results [74], making diagnosis difficult.

CFNDD is related to malabsorption of nutrients either as a direct result of membrane dysfunction or indirectly via reduced digestive enzyme availability and pancreatic dysfunction due to abnormal ion transport [75]. With nutritional intervention, CFNDD should subside.

Although data are limited, CF does appear to be associated with atopy (but not AD) and increased risk of cutaneous drug reactions [75].

### 8.2. Phenylketonuria

After the first few months of birth, patients with PKU may present with severe acute dermatitis on most of the body. Individuals may also be photosensitive, hypopigmented relative to siblings and parents, and present with a “musty odor” in sweat and urine due to a buildup of phenylalanine metabolic byproducts [76]. The severities of other symptoms depend on age and generally include intellectual disability, microcephaly, seizures, and movement disorders.

### 8.3. Zinc Deficiency and Biotin Deficiency

Most cases of ZD occur in premature infants who are still breast feeding at three months of age when zinc levels in mother’s milk drop as the infant’s zinc requirements increase. Typical presentation includes a “horseshoe” pattern of facial dermatitis or crusty erosions on the cheeks and chin. “Diaper area” involvement typically features sharply demarcated plaques with peripheral scale that, unlike Candidiasis, classically spares the folds. In older children, lesions localize to the extensor surfaces of the elbows and knees. Paronychia, alopecia, diarrhea, and recurrent skin infections are also common. Children with ZD tend to be irritable although there is no xerosis or pruritus. Until solid food is introduced, zinc supplementation improves clinical signs.

BD, or multiple carboxylase deficiency, is a rare autosomal recessive trait based on reduced activity of any or all three associated biotin-dependent carboxylases. BD presents with alopecia and maculopapular erythemic rash around the eyes, nose, mouth, ears, and genitals. The eruption is often accompanied by vomiting, apnea, hypotonia, seizures, lethargy, recurrent infections, metabolic acidosis, organic aciduria, hyperammonemia, and difficulty feeding. Symptoms generally present within the first year of life. Family history of BD is an important risk factor. Biotin supplementation improves symptoms.

### 8.4. Food Allergy

Cutaneous reactions are the most common presentation of food allergy/intolerance in children and can manifest as AD, allergic or irritant CD (see Section 2.4), or DH.

#### Dermatitis Herpetiformis

Although gastrointestinal symptoms may not be present, DH is directly related to gluten-sensitive enteropathy (GSE); however, the severity of DH does not appear to be related to the severity of intestinal inflammation [77]. DH most often presents as symmetrical circular groupings of polymorphic erythremic papulovesicles on extensor surfaces of the limbs, back, or buttocks; however, due to intense pruritus (which sometimes precedes lesion development), excoriations, and crusting may be evident instead. The face and scalp are sometimes affected and urticaria may be present. Lichenification and hypopigmentation may develop due to chronic lesions and scratching.

Prevalence of DH is greater in Caucasians [78]. Prepubertal children generally manifest GSE as celiac disease characterized by gastrointestinal symptoms, while in early to middle adulthood, DH is more common. Diagnosis requires a high index of suspicion and is confirmed by observation of granular IgA deposits along the dermal-epidermal border via direct immunofluorescence. Oral corticosteroids have little effect, but potent-to-very potent TCS may help alleviate pruritus [78].

## 9. Other Differential Diagnoses

### 9.1. Graft-versus-Host Disease

The most common clinical manifestation of acute GVHD (occurring within 100 days of transplant) is a pruritic or tender maculopapular rash usually involving the palms, soles, neck, ears, and/or shoulders, which may progress to involve the whole body [79,80]. Findings are often subtle but severe cases may progress to erythroderma, bullae, or desquamation and, rarely, epidermal necrolysis. Mucositis and hepatic and/or gastrointestinal signs and symptoms support the diagnosis. Similar symptoms of acute GVHD may develop in infants with SCID (see Section 7.3) due to engraftment by transplacentally acquired maternal T cells (maternal-fetal GVHD) [81].

### 9.2. Drug Eruptions

**Figure 25:**
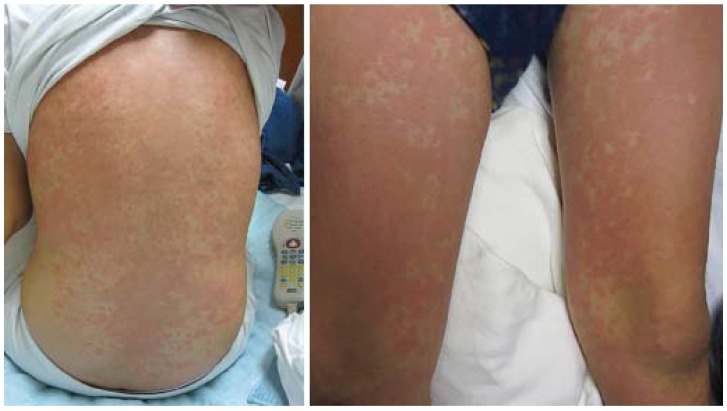
Drug eruption.

Drug eruptions (DE), are the most common rashes managed by physicians [57]. Most frequently, DE manifest as erythema, urticaria, erythema multiforme (EH-like reaction), or fixed drug eruption. Erythematous lesions may be maculopapular or morbilliform, and generally begin on the trunk and are symmetrically distributed (Figure 25). Urticaria may be an immediate- or delayed-type reaction, and presents with small papules to large annular plaques that are often pruritic. Fixed drug eruptions are solitary or multiple eruptions that occur in the same location with each drug exposure and may include central blisters, which are rarely pruritic.

DEs can mimic a wide range of dermatoses. Most are diagnosed from medical history and clinical examination, but skin biopsy may be helpful. DE should be considered in any patient presenting with cutaneous symptoms without prior history of skin disease, including AD.

Treatment is supportive and primarily includes cessation of the drug, if possible. Dermatitis can be treated with TCS.

## 10. Distinguishing Features

Differentiating AD from other potential diagnoses can be difficult. Not only are there a number of “imitators” with similar clinical features, but there are several diagnoses that can coexist with and/or complicate AD.

There is no definite laboratory test for AD. Patch testing (to diagnose CD), bacterial culture (to diagnose impetigo), microscopy (to diagnose tinea, scabies), or biopsy and other laboratory parameters such as cell flow cytometry (to diagnose CTCL) may be helpful, but in most cases, diagnosis is based on clinical signs only. In our clinical experience, several key features distinguish AD from other diagnoses (Table 4).

## 11. Conclusions

Although many differential diagnoses have been discussed, few are as common as AD. Age of onset, presence of xerosis and/or pruritus, distribution and appearance of lesions, and accompanying symptoms are key to successful diagnosis. Clinicians should keep in mind that inflammatory skin diseases can coexist (“overlap”) and may be complicated by or predispose patients to infections and infestations. Keratosis pilaris and ichthyosis vulgaris may be present as a minor feature of AD, or may be present outside the context of AD. Dermatitis is associated with serious immunodeficiency disorders, ichthyoses, malignancies, nutritional disorders, and other conditions (graft-*versus*-host disease, drug eruptions), and should not be overlooked.

**Table 4:** Distinguishing clinical features of most prominent differential diagnoses. [blank] indicates rare/unusual; **✓**, sometimes; **✓**^+^, often/usually; **✓**^++^, always/nearly always/most often; Δ, variable; –, not applicable; AD, atopic dermatitis; ARCI, autosomal recessive congenital ichthyosis; CS, corticosteroids; CTCL, cutaneous T-cell lymphoma; TCS, topical corticosteroids; XLRI, X-linked recessive ichthyosis; ^a^ Concurrent disease may be observed (*i.e.*, “overlap”).

	History		Signs/Symptoms			Distribution	Other Distinguishing/Diagnostic Features
	Infantile Onset	Associated with AD	Pruritus/Excoriations	Xerosis	Well Circumscribed Lesions		
Atopic Dermatitis	✓^++^	–	✓^++^	✓^++^		infants: face, trunk, extensor extremities	chronic with intermittent flares
						children: flexors	
						adults: hands	
						all ages: spares “diaper area”/groin, axilla	
Seborrheic Dermatitis ^a^	✓^++^				✓	scalp, face, skin folds	coarse, greasy, yellow scale
Psoriasis ^a^			✓	✓	✓^++^	extensors, scalp, “diaper area”; “Köebnerizes”	thick, silvery-white scaling
Nummular Dermatitis ^a^		✓	✓	✓^+^	✓^++^	extremities	very TCS-responsive
Irritant Contact Dermatitis	✓	✓^+^	✓^+^	✓^+^	✓^+^	geographic, often asymmetric	history of irritant exposure
Allergic Contact Dermatitis ^a^	✓		✓^++^	✓	✓^+^	often bilaterally symmetric	history of allergen exposure
Dermatographism		✓^+^	✓^++^		✓^+^	“Köebnerizes”	clinical response to mechanical stimulation
Pityriasis Alba		✓^+^		✓^+^	✓^+^	face, trunk, extremities	poorly circumscribed, hypopigmented lesions; nonpruritic
Impetigo	✓^+^	✓			✓^+^	face, trunk, extremities	honey-colored crust, tenderness
Secondary Syphilis				✓^+^	✓^+^	trunk, extremities	mucous membrane and palmoplantar involvement
Molluscum Dermatitis	✓^+^	✓^+^	✓^+^	✓^+^		face, trunk, extremities	frequently localized; umbilicated papules (often subtle)
Eczema Herpeticum/Vaccinatum	✓^+^	✓^+^		✓^+^	✓^+^	face, AD-affected areas	“punched out” lesions
Eczema Coxsackium	✓^+^	✓^+^		✓^+^	✓^+^	AD-affected areas	acute onset, fever, subsequent onychomadesis
Viral Exanthem	✓^++^		✓	✓		face, trunk extremities	acute onset, associated constitutional symptoms
Tinea			✓	✓	✓^++^	scalp, face, trunk, extremities	trailing edge scale, lymphadenopathy, hair loss
Candidiasis	✓^++^		✓	✓	✓	skin folds, genitalia	paronychia and/or thrush
Scabies	✓^+^		✓^++^		✓	infants: palms, soles, face, scalp adults: finger webs, wrists, periareolar, genitals	visible burrows, palmoplantar pustules
Letterer-Siwe Disease	✓^++^				✓	skin fold and scalp accentuation	telangiectatic, hepatosplenomegaly
Early-Stage CTCL					✓	trunk, extremities	symptoms accompanied by weight loss or malaise
Keratosis Pilaris	✓	✓^+^	✓	✓^+^		cheeks, extensor extremities	follicular keratotic papules, underlying macular erythema
Ichthyosis Vulgaris	✓^+^	✓^+^	✓	✓^++^		spares popliteal and antecubital fossae	plate-like scale, palmoplantar hyperlinearity, non-responsive to CS
XLRI (males only)	✓^++^		✓	✓^++^		extensor surfaces, neck, face, trunk, buttocks	grey adherent scale
ARCI	✓^++^		✓	✓^++^		face, trunk, extremities	“collodion baby”
Immunodeficiency Disorders	✓^++^	✓	Δ	Δ		variable	laboratory confirmation
Nutritional Disorders	✓		✓	Δ	✓	variable	laboratory confirmation
Acute Graft-*versus*-Host Disease	✓			✓^++^		face, trunk, extremities	oral mucous membrane/palmar involvement
Drug Eruptions			✓	✓	✓	face, trunk, extremities	clinico-pathological correlations
